# Structural insights into inhibitor regulation of the DNA repair protein DNA-PKcs

**DOI:** 10.1038/s41586-021-04274-9

**Published:** 2022-01-05

**Authors:** Shikang Liang, Sherine E. Thomas, Amanda K. Chaplin, Steven W. Hardwick, Dimitri Y. Chirgadze, Tom L. Blundell

**Affiliations:** 1 Department of Biochemistry, University of Cambridge, Cambridge, UK; 2 Cryo-EM Facility, Department of Biochemistry, University of Cambridge, Cambridge, UK; 3 Present Address: Department of Pathology, University of Cambridge, Cambridge, UK; 4 Leicester Institute for Structural and Chemical Biology, Department of Molecular and Cell Biology, University of Leicester, Leicester, UK

**Keywords:** Cryoelectron microscopy, Drug development, Kinases, Small molecules

## Abstract

The DNA-dependent protein kinase catalytic subunit (DNA-PKcs) has a central role in non-homologous end joining, one of the two main pathways that detect and repair DNA double-strand breaks (DSBs) in humans^1,2^. DNA-PKcs is of great importance in repairing pathological DSBs, making DNA-PKcs inhibitors attractive therapeutic agents for cancer in combination with DSB-inducing radiotherapy and chemotherapy^3^. Many of the selective inhibitors of DNA-PKcs that have been developed exhibit potential as treatment for various cancers^4^. Here we report cryo-electron microscopy (cryo-EM) structures of human DNA-PKcs natively purified from HeLa cell nuclear extracts, in complex with adenosine-5′-(γ-thio)-triphosphate (ATPγS) and four inhibitors (wortmannin, NU7441, AZD7648 and M3814), including drug candidates undergoing clinical trials. The structures reveal molecular details of ATP binding at the active site before catalysis and provide insights into the modes of action and specificities of the competitive inhibitors. Of note, binding of the ligands causes movement of the PIKK regulatory domain (PRD), revealing a connection between the p-loop and PRD conformations. Electrophoretic mobility shift assay and cryo-EM studies on the DNA-dependent protein kinase holoenzyme further show that ligand binding does not have a negative allosteric or inhibitory effect on assembly of the holoenzyme complex and that inhibitors function through direct competition with ATP. Overall, the structures described in this study should greatly assist future efforts in rational drug design targeting DNA-PKcs, demonstrating the potential of cryo-EM in structure-guided drug development for large and challenging targets.

## Abstract (web summary)

Cryo-electron microscopy structures of DNA-dependent protein kinase catalytic subunit bound to ATPγS and four inhibitors (wortmannin, NU7441, AZD7648 and M3814) provide molecular details and insights useful for drug design.

## Main

DNA double-strand breaks (DSBs) are the most toxic form of DNA damage. Two major pathways, homologous recombination (HR) and non-homologous end joining (NHEJ), repair DSBs^1^. HR requires DNA end resection and is active during the S and G2 phases of the cell cycle, when a sister chromatid is available as a repair template^5^. By contrast, NHEJ directly ligates DNA ends efficiently in the absence of any template^2^.

DSBs lead to increased genome instability and trigger cell death. This is widely exploited in the treatment of cancer in both radiotherapy using ionizing radiation (IR) and chemotherapy using topoisomerase II inhibitors^2,3^. However, intrinsic DNA damage response and repair provide tumour cells with some protection. The pathological DSBs caused by IR and topoisomerase II inhibitors are mainly repaired by NHEJ, which requires DNA-dependent protein kinase (DNA-PK), comprising the DNA-dependent protein kinase catalytic subunit (DNA-PKcs) and a Ku70–Ku80 heterodimer^6–8^. DNA-PKcs, which belongs to the phosphoinositide 3-kinase (PI3K)-related protein kinase (PIKK) family, has a central role in the regulation of NHEJ^9^. In combination with IR or genotoxic chemotherapy, inhibition of DNA-PK kinase activity can improve cancer therapy^4^. There have been many efforts to develop small-molecule inhibitors targeting the ATP-binding site of DNA-PKcs, informed by early studies of synthetic small-molecule PI3K inhibitors^10^. The older-generation DNA-PK inhibitors, including CC115, KU-0060648, LY294002, LY3023414, NU7026 and NU7441, are effective, but all have different limited selectivity against PI3K and PIKK members (especially mTOR and PI3Kγ). The newer-generation inhibitors, developed from large-scale screening, include VX-984, M3814 and AZD7648, which have better selectivity for DNA-PKcs^4,10,11^.

So far, to our knowledge, no structures have been published of DNA-PKcs in complex with ATP or any inhibitor. This has limited understanding of both ATP binding to DNA-PKcs and the mode of action of the inhibitors, including drug candidates, and has posed a major hurdle to lead development. Here we report structures of DNA-PKcs in complex with adenosine-5′-(γ-thio)-triphosphate (ATPγS) and other inhibitors, including the broad-spectrum PI3K inhibitor wortmannin, the older-generation DNA-PKcs selective inhibitor NU7441 and the newer-generation AZD7648 and M3814, which are in clinical trials. The structures allow understanding of DNA-PKcs binding to ATP before substrate binding and catalysis, the modes of action of the inhibitors and the mechanisms by which they achieve specificity. They also provide structural guidance for future drug design targeting DNA-PKcs, demonstrating the great potential of cryo-EM in structure-based drug discovery.

## DNA-PKcs in complex with ATPγS–(Mg^2+^)_2_

**Fig. 1:**
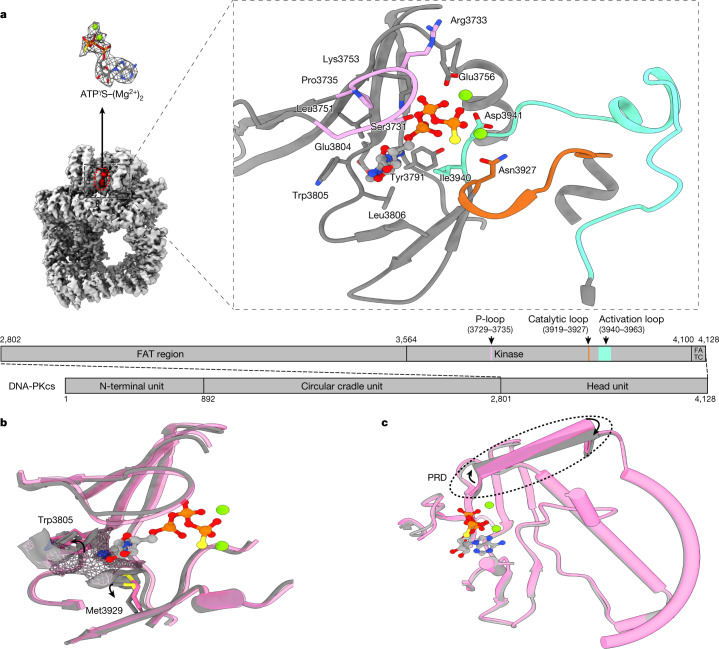
ATPγS–(Mg^2+^)_2_ interaction with and regulation of DNA-PKcs. **a**, Coulomb potential map of the DNA-PKcs–ATPγS–(Mg^2+^)_2_ complex. The expanded view shows ATPγS–(Mg^2+^)_2_ binding in the ATP-binding groove. ATPγS (light grey), together with two Mg^2+^ ions (fluorescent green), coordinates the N- and C-lobes, especially the p-loop (plum), catalytic loop (chocolate) and activation loop (azure) of DNA-PKcs (grey). The γ-phosphate group points towards the substrate-binding site. The top left image shows the clear Coulomb potential map for modelling of ATPγS–(Mg^2+^)_2_, while the schematic representation below highlights the three units of DNA-PKcs and detailed composition of the head unit. **b**, Opening of the ATP-binding groove entrance. The residues on both sides of the ATP-binding groove entrance, Trp3805 and Met3929, exhibit an outward rotation that allows docking of the adenine moiety of ATPγS. Apo DNA-PKcs (Protein Data Bank (PDB), 6ZFP) is coloured pink with a mesh surface. **c**, ATPγS–(Mg^2+^)_2_ regulation on the PRD. PRD blocks the substrate-binding site. When ATP binds, PRD is tilted and moves away from its position in the apo structure.

**Extended Data Fig. 1:**
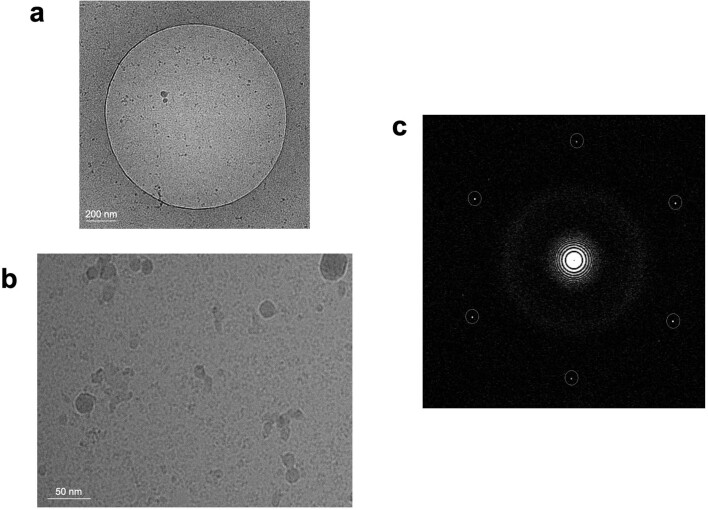
Home-made graphene oxide grid for DNA-PKcs/ ATPγS-Mg^2+^_2_ complex. **a**, TEM image of grid hole with suspended graphene oxide on. **b**, Cryo-EM micrograph of DNA-PKcs/ ATPγS-Mg^2+^_2_ complex. **c**, Fourier transform of b with the graphene oxide reciprocal lattice circled in white dashed line. The graphene-oxide grid is replicated for the DNA-PKcs/complex studies.

**Extended Data Fig. 2:**
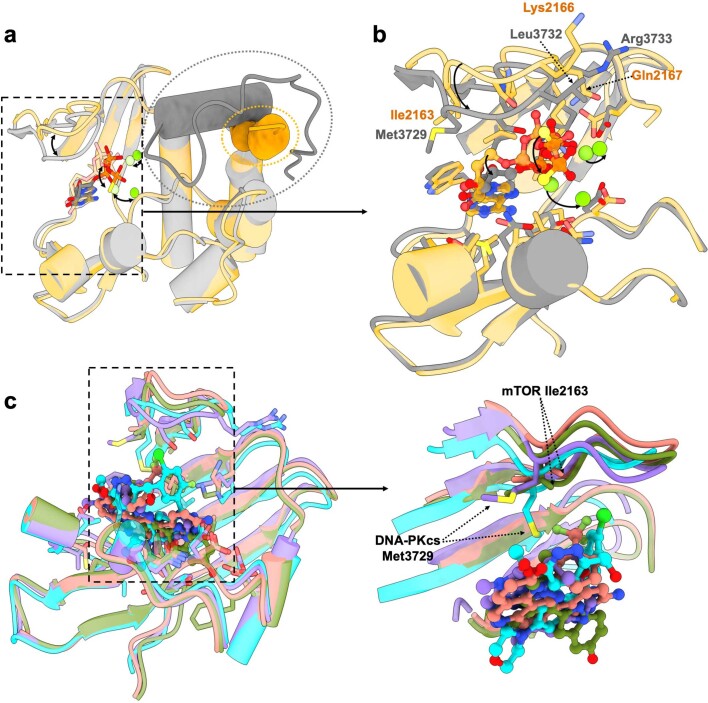
Comparisons between DNA-PKcs and mTOR in complex with ligands. **a**, Comparison of ATPγS-Mg^2+^_2_ binding in the ligand binding grooves of DNA-PKcs and mTOR (PDB entry: 4JSP)^12^. The PIKK regulatory domain (PRD) of mTOR is lifted and removed completely from the substrate-binding site while the PRD of DNA-PKcs still docks there, although it is lifted compared to apo DNA-PKcs. DNA-PKcs/ATPγS-Mg^2+^_2_ complex is coloured grey while mTOR/ATPγS-Mg^2+^_2_ is coloured orange. PRDs of DNA-PKcs and mTOR are circled in dashed lines. **b**, Close-up view of the ligand-binding pocket of DNA-PKcs and mTOR. Residues contributing to the pocket are highly conserved between DNA-PKcs and mTOR. The major differences lie in the p-loop, including residue composition and loop conformation. The residues that differ are labeled in the figure. Compared to that of mTOR, the p-loop of DNA-PKcs is positioned more inward and closer to the C-lobe, leaving a narrower channel for the ligands to enter. Moreover, DNA-PKcs p-loop pushes the ligands more toward the C-lobe as the ribose moiety and the phosphate groups of ATPγS-Mg^2+^_2_ rotate towards the C-lobe compared to those of mTOR. The adenine moieties overlay well as the deep hydrophobic pockets are highly structurally similar. **c**, Comparison of mTOR in complex with specific inhibitor Torin2 (PDB code: 4JSX) and pp242 (PDB code: 4JT5) and DNA-PKcs in complex with specific inhibitor AZD7648 and M3814 and close-up view. The major differences are in the p-loop conformations. Moreover, among the few residues that differ, Met3729 of DNA-PKcs is more extended compared to the Ile2163 of mTOR at the equivalent position and acts as the gatekeeper. Moreover, it affects the conformation of DNA-PKcs p-loop when different ligands bind. DNA-PKcs in complex with AZD7648 is coloured purple, DNA-PKcs in complex with M3814 is coloured cyan, mTOR in complex with Torin2 is coloured salmon and mTOR in complex with pp242 is coloured olive.

DNA-PKcs purified from HeLa cell nuclear extract was incubated with ATPγS and loaded onto a previously prepared grid with a support film of homemade single-layer graphene oxide (Extended Data Fig. 1). The overall resolution of the DNA-PKcs–ATPγS complex is 3.40 Å with the local resolution of the catalytic core region better than 3 Å, showing clear density for the modelling of ATPγS with two Mg^2+^ ions and demonstrating a similar structure to that of the homologue mTOR (Fig. 1a and Extended Data Fig. 2a)^12^. The p-loop (residues 3729–3735) interacts closely with the phosphate groups of ATPγS. Central to this is the interaction between the β-phosphate and the main chain NH group of Arg3733, together with the side chain of Ser3731. Lys3753 interacts with the α-phosphate of ATPγS. Asn3927, Asp3941 and Glu3756 together coordinate the Mg^2+^ ions. The γ-phosphate of ATPγS points towards the substrate-binding site. The ‘hinge loop’ between the N-lobe and C-lobe, together with the two lobes, constitutes a hydrophobic surface, formed by the side chains of Leu3751, Tyr3791, Trp3805, Leu3806 and Ile3940 and the main chain of Glu3804, to which the adenine moiety of ATPγS can bind. Thus, ATPγS and the Mg^2+^ ions bind in the ATP-binding groove of DNA-PKcs, coordinating the N- and C-lobes, including the p-loop, activation loop and catalytic loop of the kinase. Conformational changes include the outward rotation of both Trp3805 and Met3929, opening the pocket to accommodate ATP (Fig. 1b)^13^, and coordinated movements of α-helices in the head unit of the protein (Fig. 1c). Notably, the PRD (residues 4009–4039) does not interfere with the interaction of DNA-PKcs with ATP and Mg^2+^. However, the α-helix of the PRD (residues 4009–4023) of DNA-PKcs is moved away from the substrate-binding site following ATP binding, partly relieving the blockage by PRD of the substrate-binding site required for subsequent catalysis (Fig. 1c).

## DNA-PKcs in complex with inhibitors

**Fig. 2:**
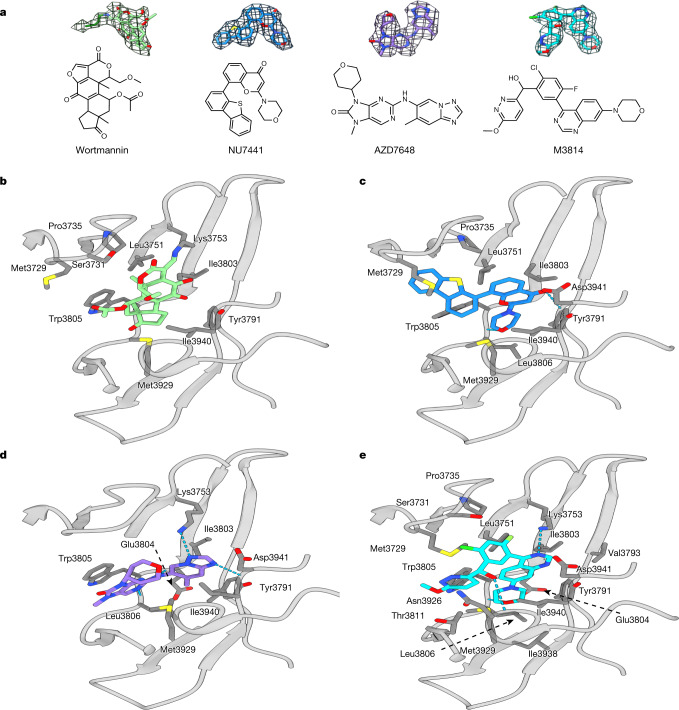
ATP competitive inhibitors (wortmannin, NU7441, AZD7648 and M3814) and their modes of binding to DNA-PKcs. **a**, Inhibitors investigated and their corresponding Coulomb potential maps. **b**, Binding of wortmannin (green) to the ATP-binding site, where it is covalently modified by the primary amine group of Lys3753. **c**, Binding of NU7441 (blue) to the ATP-binding site. **d**, Binding of AZD7648 (purple) to the ATP-binding site. **e**, Binding of M3814 (cyan) to the ATP-binding site. DNA-PKcs is shown in grey.

DNA-PKcs was incubated with several DNA-PKcs inhibitors with different specificities (wortmannin, NU7441, AZD7648 and M3814), to investigate protein–ligand interactions (Supplementary Table 1). The overall resolutions of the structures range from 2.96 Å to 3.33 Å. In all the structures, the kinase core has the highest local resolution, allowing the inhibitors to be unequivocally modelled (Fig. 2a).

**Extended Data Fig. 3:**
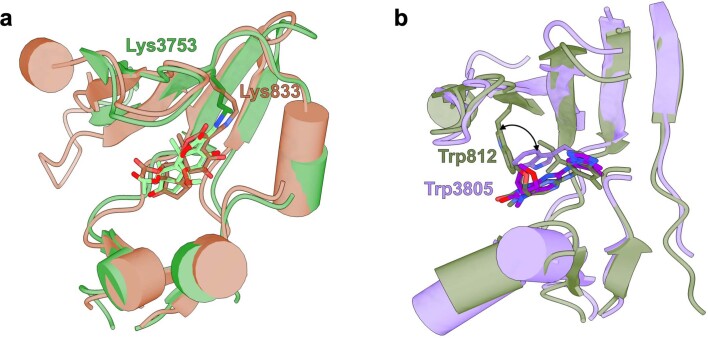
ATP competitive inhibitors in complex with homologues of DNA-PKcs. **a**, Comparison of wortmannin binding on the ATP binding groove of porcine PI3Kγ (PDB entry: 1E7U) and DNA-PKcs^15^. The Lys 833 of porcine PI3Kγ and Lys 3753 of DNA-PKcs react with the furan ring of wortmannin to form a covalent C-N bond and irreversibly inhibit the kinase activity. **b**, Comparison of AZD7648 binding on ATP binding groove of PI3Kγ (PDB entry: 6T3C) and DNA-PKcs^17^. The 90° rotation of the indole ring from PI3Kγ Trp 812 to DNA-PKcs Trp 3805 provides a better surface and strong stacking effect for the binding of AZD7648. The porcine PI3Kγ/Wortmannin complex is coloured brown. The PI3Kγ/AZD7648 complex is coloured olive.

Wortmannin is one of the early DNA-PK inhibitors used for kinase inhibition^14^. In the cryo-EM structure, wortmannin packs on one side, mainly against the N-lobe of DNA-PKcs (Leu3751, Ile3803 and Trp3805), while on the other side it packs against the C-lobe (Met3929 and Asp3940) (Fig. 2b). One edge of the compound is facing the deep pocket of the ATP-binding site, and the opposite edge is exposed to solvent. The primary amine (ε-amino group) of Lys3753 forms a covalent C–N bond with C19 in the furan ring of wortmannin (Fig. 2a), irreversibly inhibiting kinase activity. The ATP-binding site of DNA-PKcs has close structural complementarity to wortmannin. The C14 methyl resides in a pocket formed by Leu3751, Ile3803 and Trp3805, while the C10 methyl fits into a pocket formed by Met3729, Pro3735 and Leu3751 (Fig. 2b). There is no density corresponding to the acetoxy group attached to C11 of wortmannin, as was observed in the published structure of wortmannin in complex with porcine PI3Kγ (Extended Data Fig. 3a)^15^.

Compared with wortmannin, NU7441 is a more selective inhibitor, developed from the earlier inhibitors NU7026 and LY294002, which were derived from the broad-spectrum protein kinase flavonoid inhibitor quercetin^16^. The chromone core and morpholino ring of NU7441 bind in the centre of the deepest pocket formed by Leu3751, Tyr3791, Ile3803, Trp3805, Leu3806, Ile3940 and Met3929, within which O1 and O27 of NU7441 form hydrogen bonds with the peptide backbones of Asp3941 and Leu3806 (Fig. 2c). The dibenzothiophene group interacts with the N-lobe and docks in the hydrophobic groove of Met3729, Pro3735 and Leu3751 (Fig. 2c).

AZD7648 is a recently developed DNA-PK inhibitor shown to be highly selective and to enhance the efficacy of doxorubicin and IR^11,17^. Moreover, AZD7648 was identified as a potential combinational therapy with the PARP inhibitor olaparib and is currently under clinical trial for advanced malignancies (trial identifier, NCT03907969)^11^. The developers of AZD7648 took advantage of a previously published crystal structure of AZD7648 bound to PI3Kγ^17^. In the DNA-PKcs–AZD7648 complex, the compound binds the hinge loop (Fig. 2d). The triazolopyridine moiety with a methyl group lies in the deep hydrophobic pocket formed by Tyr3791, Ile3803, Leu3806 and Ile3940. The purine moiety docks in the narrow tunnel formed by Trp3805, Leu3806 and Met3929. Similarly to the PI3Kγ–AZD7648 complex, the N3 hydrogen of AZD7648 (the aniline NH) forms bonds with the backbone oxygen of Glu3804 and N7 accepts the hydrogen from the backbone NH group of Leu3806 (ref. ^17^). N6 in the triazolopyridine moiety also binds to the backbone NH group of Asp3941. There is a further hydrogen bond in the case of DNA-PKcs–AZD7648: N5 forms a bond with the primary amine group of Lys3753. Comparison of the two structures shows a 90° rotation of the indole ring for Trp812 in PI3Kγ relative to Trp3805 in DNA-PKcs, which in DNA-PKcs not only provides a better hydrophobic binding surface but also aligns nicely to the AZD7648 purine ring, leading to a π stacking interaction that enhances affinity (Extended Data Fig. 3b).

M3814 (peposertib or nedisertib) is another recently developed DNA-PK selective inhibitor^18,19^. Preclinical studies have revealed its synergy with radiotherapy and chemotherapies using doxorubicin and etoposide^20^. The compound was shown to be well tolerated with modest efficacy in unselected tumours in phase 1 clinical trial results (trial identifier, NCT02316197)^19^; M3814 is currently in four phase 2 clinical trials targeting different cancers (trial identifiers, NCT03770689, NCT04068194, NCT04071236 and NCT04172532). According to our structure, the quinazoline and morpholino moieties of the compound fit well into the deepest, largely hydrophobic pocket formed by the side chains of Leu3751, Tyr3791, Val3793, Ile3803, Trp3805, Leu3806, Ile3938 and Ile3940 and the main chains of Glu3804 and Asp3941 (Fig. 2e). The chloro-fluorobenzene ring rotates by ~60° and points towards the N-lobe, facing the side chains of Met3729, Ser3731, Pro3735, Leu3751 and Lys3753. The remaining moiety containing the pyridazine ring then rotates back to be almost parallel to the quinazoline plane, lying in the groove of Met3729, Trp3805, Thr3811, Asn3926 and Met3929.

## Comparison of binding modes

**Fig. 3:**
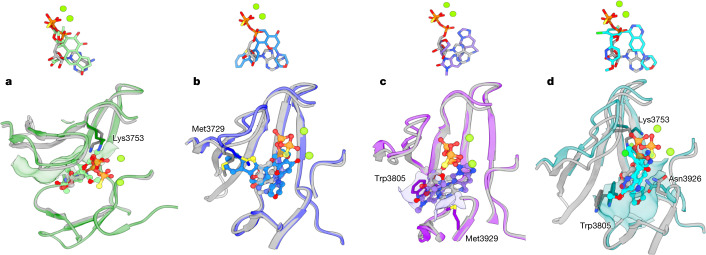
Comparisons of the binding modes among ATPγS–(Mg^2+^)_2_ and the four inhibitors. **a**, Comparison of the binding modes of ATPγS–(Mg^2+^)_2_ and wortmannin in DNA-PKcs. Top, binding conformations of ATPγS–(Mg^2+^)_2_ (light grey, ATPγS; fluorescent green, Mg^2+^ ions) and wortmannin (green). Bottom, conformational differences in the binding groove of DNA-PKcs between ATPγS–(Mg^2+^)_2_ (grey) and wortmannin (green). **b**, Comparison of the binding modes of ATPγS–(Mg^2+^)_2_ and NU7441 in DNA-PKcs. Top, binding conformations of ATPγS–(Mg^2+^)_2_ and NU7441 (blue). Bottom, conformational differences in the binding groove of DNA-PKcs between ATPγS–(Mg^2+^)_2_ and NU7441 (blue). **c**, Comparison of the binding modes of ATPγS–(Mg^2+^)_2_ and AZD7648 in DNA-PKcs. Top, binding conformations of ATPγS–(Mg^2+^)_2_ and AZD7648 (purple). Bottom, conformational differences at the binding groove of DNA-PKcs between ATPγS–(Mg^2+^)_2_ and AZD7648 (purple). **d**, Comparison of the binding modes of ATPγS–(Mg^2+^)_2_ and M3814 in DNA-PKcs. Top, binding conformations of ATPγS–(Mg^2+^)_2_ and M3814 (cyan). Bottom, conformational differences at the binding groove of DNA-PKcs between ATPγS–(Mg^2+^)_2_ and M3814 (cyan).

All the inhibitors studied target the ATP-binding groove of DNA-PKcs, overlapping with the ATPγS-binding site (Fig. 3). Among them, wortmannin has the maximum overlap. Compared with ATPγS, wortmannin has greater complementarity to the binding site (Fig. 3a), binding deeper into the ATP adenine-moiety pocket, with its two protruding methyl groups fitting better into the hydrophobic pocket on the surface of the N-lobe.

NU7441 binds quite differently from ATPγS to the ATP-binding groove. The chromone core and the morpholino ring of NU7441 bind to the pocket occupied by the adenine moiety and α-phosphate group of ATPγS (Fig. 3b). The dibenzothiophene moiety overlaps with the ATPγS ribose and inserts into the groove between the p-loop and the hinge loop, which is formed by the 120° inward swinging of Met3729.

In the case of AZD7648, the triazolopyridine moiety with a methyl group docks deeper than the adenine moiety of ATPγS in the same pocket (Fig. 3c). While the tetrahydropyran ring occupies the same space as the ATPγS ribose, the purine ring of AZD7648 fits nicely into the empty groove between Trp3805 and Met3929.

M3814 has the least overlap with ATPγS in the binding groove. The quinazoline and morpholine parts reach deeper into the pocket of the adenine moiety and α-phosphate group of ATPγS (Fig. 3d). Moreover, when the compound binds, the side chain of Trp3805 moves inwards, changing the open adenine-binding site to an enclosed hydrophobic pocket, in which the morpholine ring of M3814 is fully accommodated. The chloro-fluorobenzene ring overlaps with the ATPγS α-phosphate and points towards the N-lobe, causing an uplift of the p-loop and the region around Lys3753. The pyridazine ring is adjacent to the ATPγS ribose but closer to the C-lobe, where Asn3926 rotates towards M3814 to form a small groove for docking of the drug.

**Fig. 4:**
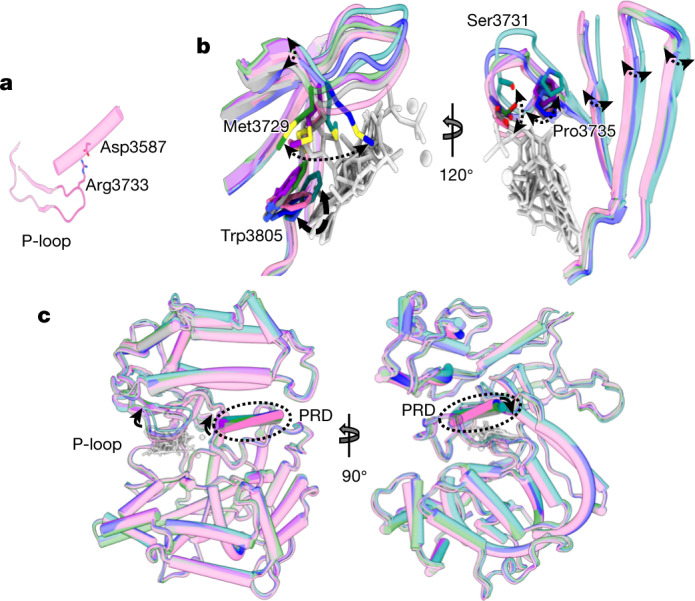
Conformational changes resulting from binding of ATPγS–(Mg^2+^)_2_ and competitive inhibitors. **a**, The p-loop conformation in apo DNA-PKcs (pink) is fixed by the flanking β-sheets and the electrostatic interaction between Arg3733 and Asp3587. **b**, Two views, related by rotation of 120^o^, of the effect of binding different ligands on the conformation of the p-loop. These conformational changes resemble the movement of a spring leaf. The corresponding movement of the flanking β-sheets transmits a conformational change to the core DNA-PKcs kinase region. Grey, ATPγS; green, wortmannin; blue, NU7441; purple, AZD7648; cyan, M3814. **c**, Orthogonal views of the p-loop conformations regulating the conformation of the DNA-PKcs kinase region, including the PRD.

The different inhibitors result in varying conformational changes that each modify the surface properties to optimize binding interactions with the ligand. There are two key regions of conformational change. One is the p-loop, where the conformation is guided by the residues with their side chains facing the ATP-binding groove. In apo DNA-PKcs, the position of the p-loop is restrained by the flanking β-sheets and the electrostatic interaction between Arg3733 and Asp3587 (Fig. 4a). The conformations of Met3729, Ser3731 and Pro3735 change according to the different chemical properties of the ligands, resulting in an up–down movement of the p-loop like a ‘spring leaf’. This movement induces corresponding conformational changes in the flanking β-sheets that are passed on to the hydrophobic core of the DNA-PKcs head region (Fig. 4b). The resulting conformational changes of DNA-PKcs, especially the movement of the PRD, appear concerted (Fig. 4c). The other key region of conformational change is at Trp3805 on the hinge loop, which guards the entrance to the main pocket (Fig. 4b). The indole side chain of Trp3805 contributes to the stacking effects and hydrophobic interactions with ligands, and its movement impacts the architecture of the binding site.

## Ligand regulation and future development

**Extended Data Fig. 4:**
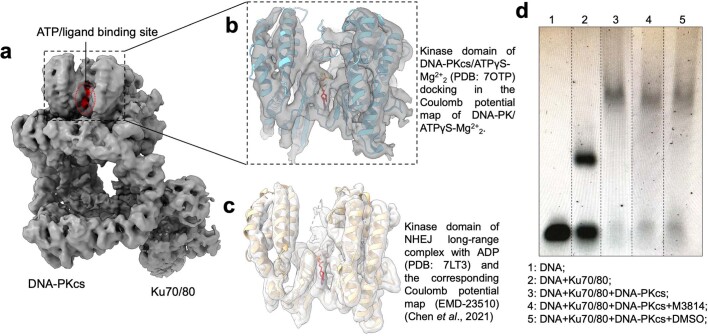
Ligand binding in the same pocket of DNA-PKcs in higher-order complexes without structurally affecting the assemblies. **a**, The Coulomb potential map of DNA-PK holoenzyme in complex with ATPγS-Mg^2+^_2_. **b**, Close-up view of the DNA-PKcs kinase domain in the DNA-PK/ ATPγS-Mg^2+^_2_ Coulomb potential map with the model of the kinase domain of the DNA-PKcs/ ATPγS-Mg^2+^_2_ complex docked in. **c**, Close-up view of the Coulomb potential map of NHEJ long-range complex (EMD-23510) and the related kinase domain model (PDB: 7LT3), which includes the ligand binding of ADP^24^. **d**, EMSA/gel shift assay of Ku70/80, DNA-PKcs and DNA-PKcs inhibitor with DNA (replicated three times). For gel source data, see Supplementary Figure 1.

Understanding of the mechanism of DNA-PKcs in DNA repair has recently been advanced through structural studies using crystallography and cryo-EM^13,21–26^. However, technical challenges and limitations have been the major hurdle in biochemical and structural investigations. The structure of DNA-PKcs–ATPγS–(Mg^2+^)_2_ described here shows how ATPγS and two Mg^2+^ ions occupy the ATP-binding groove, coordinating the p-loop, activation loop and catalytic loop. The presence of ATPγS–(Mg^2+^)_2_ results in movement of the PRD to partly relieve the blockage of the substrate-binding site before catalysis. This is consistent with the observation that DNA-PKcs can be active in vitro in the absence of assembly of the DNA-PK holoenzyme and explains why the kinase activity of the C terminus of DNA-PKcs is not stimulated by Ku70–Ku80 and DNA^27^. Together with the structures of the DNA-PKcs–inhibitor complexes, this reveals that PRD conformation can be regulated by spring leaf-like p-loop movements. Moreover, we obtained a structure for the DNA-PK–ATPγS–(Mg^2+^)_2_ complex at a medium resolution of 4.3 Å (Extended Data Fig. 4a, b). In this structure, the kinase domain of DNA-PKcs fits nicely into the corresponding region of the map of DNA-PK–ATPγS–(Mg^2+^)_2_, indicating that the binding modes of the ligands in the single polypeptide chain of DNA-PKcs and the holoenzyme are similar. Moreover, we conducted electrophoretic mobility shift assays (EMSAs) to confirm that the inhibitors do not stop formation of the DNA-PK holoenzyme (Extended Data Fig. 4d). In addition, structural and single-molecule studies have demonstrated that binding of ligands does not affect dimerization of the long-range NHEJ complex involving DNA-PKcs^24,28^. Therefore, DNA-PKcs inhibitors should not structurally disturb high-order complex formation. They function via direct ATP competition.

Current drug candidates have been successfully developed from large-scale screening targeting the ATP-binding site, but molecular details of the modes of action of such candidates have been unclear^4,10^. In our structures, all inhibitors investigated are less elongated than ATP and do not extend beyond the position of the α-phosphate group. However, the inhibitors more effectively target the deep hydrophobic pocket where the adenine moiety of ATP is located. The inhibitors all have large hydrophobic groups that bind deeply into this pocket, achieving high occupancy. It appears that the occupancy level of the pocket is related to selectivity: NU7441, AZD7648 and M3814 display higher occupancy than wortmannin. Comparisons between the older-generation inhibitor NU7441 and the newer-generation inhibitors AZD7648 and M3814 indicate that selectivity can also be improved by exploring the entrance tunnel between the N- and C-lobes and the peripheral region on the C-lobe (Fig. 3c, d). Comparison of the structures of DNA-PKcs–ligand complexes with those of mTOR–ligand complexes demonstrates that inhibitor specificity is related to the p-loop conformation and composition (Extended Data Fig. 2a–c). The ligand-binding pockets of DNA-PKcs and mTOR are highly similar, with the main differences lying in the p-loops (Extended Data Fig. 2b). In DNA-PKcs, the p-loop is closer to the C-lobe than it is in mTOR. Together with the extended side chain of Met3729, the p-loop of DNA-PKcs creates a narrower path to the binding pocket, the effect of which can be visualized through comparison of ATPγS–(Mg^2+^)_2_ binding modes (Supplementary Fig. 2a, b). While the adenine moieties overlap nicely in the two structures, the ribose moiety and phosphate groups have different binding modes due to p-loop differences (Extended Data Fig. 2b).

**Extended Data Fig. 5:**
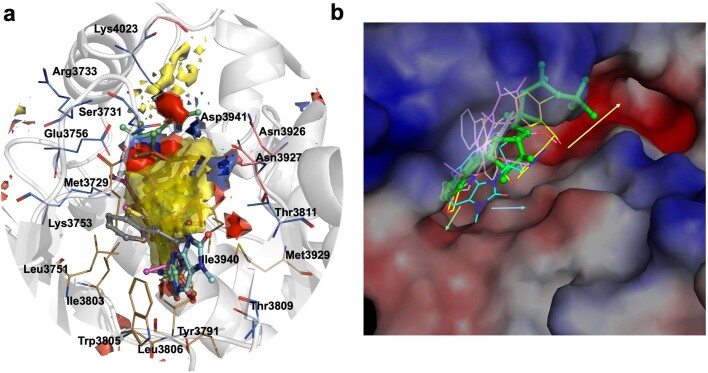
Hotspot analysis for further structure-guided inhibitor development. **a**, Hotspot map of DNA-PKcs ATP binding site contoured at 14. The apolar, donor and acceptor hotspot regions are depicted as yellow, blue and red surfaces with the key contributing residues shown as brown, salmon and blue lines respectively. ATPγS-Mg^2+^_2_ is shown in green stick-ball representation with inhibitors wortmannin (grey), NU7441 (pink), AZD7648 (blue) and M3814 (brown) overlaid. **b**, Surface electrostatic representation of DNA-PKcs in complex with ATPγS (green stick-ball) and inhibitors wortmannin (grey), NU7441 (pink), AZD7648 (blue) and M3814 (yellow) overlaid as lines. AZD7648 and M3814 are seen extending to the edge of the ATP pocket due to unique interactions with residues near the catalytic loop, not seen with the other two inhibitors. Additional lead optimization may involve elaborating compounds to utilize interactions on the catalytic loop (blue arrow) and base of C-lobe (green arrow). Growing compounds further into the groove (yellow arrow) occupied by the activation loop, thereby engaging additional polar and electrostatic contacts, allows extension further away from the ATP binding site.

Future inhibitor development can also be guided using our in-house hotspot-mapping program^29^. Hotspots can be defined as areas within the protein that make an essential contribution to the overall binding of small molecules^30^. The predicted hotspot map of the catalytic core of DNA-PKcs exhibits a large apolar region at the centre, juxtaposed with polar hydrogen-bond donor and acceptor regions (Extended Data Fig. 5a). The hydrophobic core overlaps with the adenosine moiety of ATPγS as well as the central heterocyclic scaffold of the inhibitors described in this study. Strong polar regions can be observed adjacent to this hydrophobic core, close to the p-loop, and overlap with the ATP phosphate group-binding site (Extended Data Fig. 5a). A second polar hotspot region is seen at the edge of the ATP pocket close to the catalytic loop. Of note, the more selective inhibitors examined in this study, AZD7648 and M3814, have functional groups that engage this region (Extended Data Fig. 5a, b). The polar contacts mediated by the side chain of Thr3811 and the backbone carbonyl of Asn3926 here allow compounds AZD7648 and M3814, respectively, to adopt a binding mode distinct from those of ATP, wortmannin and NU7441 by extending outside the ATP-binding groove (Extended Data Fig. 5b). It is tempting to speculate that interactions at this part of the ATP pocket, close to the catalytic loop, could further contribute to selective inhibition of DNA-PKcs in addition to the p-loop conformation. Therefore, introducing functional groups to engage Asn3926, Asn3927, Thr3811 and Thr3809 in this region of the active site may potentially help further enhance selective inhibition of DNA-PKcs.

**Extended Data Fig. 6:**
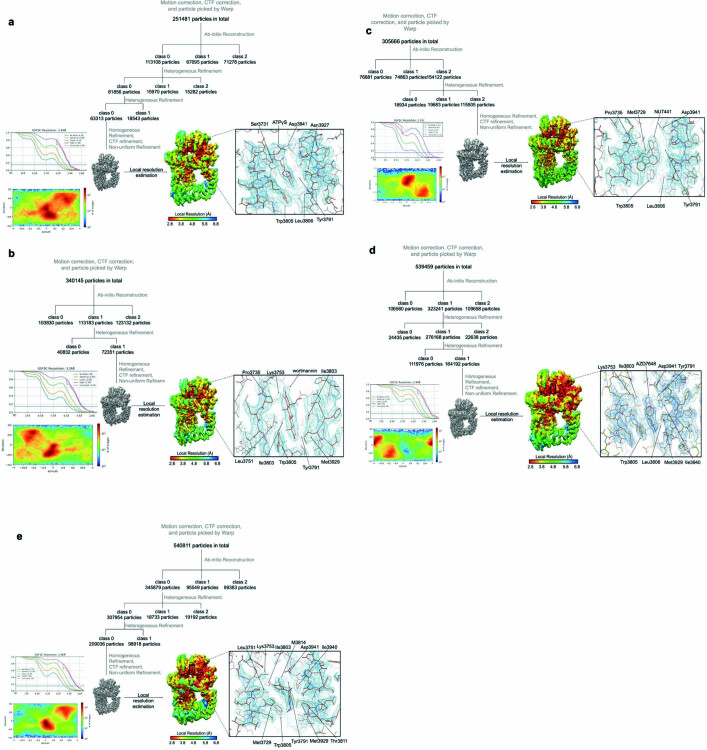
Cryo-EM data processing of the DNA-PKcs/ligand complexes. **a**, Cryo-EM data processing of the DNA-PKcs/ATPγS-Mg^2+^_2_ complex. **b**, Cryo-EM data processing of the DNA-PKcs/wortmannin complex. **c**, Cryo-EM data processing of the DNA-PKcs/NU7441 complex. **d**, Cryo-EM data processing of the DNA-PKcs/AZD7648 complex. **e**, Cryo-EM data processing of the DNA-PKcs/M3814 complex.

Furthermore, now that it is possible to obtain high-resolution structures for DNA-PKcs–ligand complexes routinely (Extended Data Fig. 6), structure-guided drug discovery can exploit other specific pockets of DNA-PKcs in the development of allosteric inhibitors or proteolysis-targeting chimeric (PROTAC) drugs with enhanced potency and selectivity^31,32^. Degradation of DNA-PKcs eliminates not only its kinase activity but also the stage provided for downstream NHEJ and DNA damage response signalling^13,32,33^. However, PROTAC drugs cannot be guaranteed to be beneficial as previous studies have shown that inhibiting kinase activity has more potent effects than knockdown or knockout^34^. Nevertheless, with optimized sample preparation and the reported cryo-EM structures, future drugs could be further developed to target allosteric sites instead of, or in addition to, the conserved kinase catalytic core, to achieve better specificity for DNA-PKcs.

## Methods

### Purification of DNA-PKcs

DNA-PKcs was purified natively from HeLa cell nuclear extracts (Ipracell) as previously described^21^. The entire purification process was conducted at 4 °C, and samples were kept on ice. Frozen pellets of nuclear extract were thawed and dialysed in precooled buffer (20 mM HEPES pH 7.6, 100 mM NaCl, 10% glycerol (vol/vol), 0.5 mM EDTA, 2 mM MgCl_2_, 5 mM DTT, 0.2 mM PMSF), and purification of DNA-PKcs was carried out in four steps of ion exchange chromatography in the sequential order of HiTrap Q, HiTrap Heparin, Mono-S and Mono-Q columns. A salt gradient of 0.1 M to 1 M NaCl was used to elute DNA-PKcs from the columns. A final gel filtration step with a Superose-6 column was used to check size, and the buffer was exchanged to the final storage buffer (20 mM HEPES pH 7.6, 200 mM NaCl, 0.5 mM EDTA, 2 mM MgCl_2_, 5 mM DTT). Purified DNA-PKcs was snap frozen in liquid nitrogen and stored at −80 °C.

### Sample preparation and cryo-EM data acquisition

DNA-PKcs was incubated on ice for 1 h with 1 mM target ligand (ATPγS, wortmannin, NU7441, AZD7648 or M3814) and then loaded onto a Quantifoil R1.2/1.3 grid with graphene oxide. For the homemade single-layer graphene oxide, the grids were previously glow discharged for 120 s at a current of 15 mA and the graphene oxide preparation was based on the previous protocol of Bokori-Brown et al.^35^. Protein samples were left to adhere for 20 s and later blotted and plunge-frozen in liquid ethane using the FEI Vitrobot Mark IV system (ThermoFisher Scientific) at 4 °C and 100% humidity. The grids were transferred to a Titan Krios electron microscope operating at a voltage of 300 kV with a K3 direct electron detector (Gatan) at the cryo-EM facility of the Department of Biochemistry, University of Cambridge. All datasets were collected in super-resolution counting mode with a magnification of ×130,000.

### Image processing and model refinement

In general, data were processed using Warp and cryoSPARC^36–38^. CTF correction, motion correction and particle autoselection were carried out using Warp. The selected particles were then subjected to cryoSPARC ab initio reconstruction to remove ice, contamination and aggregated or degraded protein components and generate the initial three-dimensional model of the complex. The nice class from ab initio reconstruction was selected and optimized through iterative rounds of heterogeneous refinement in cryoSPARC to further remove bad particles. The final optimized class of particles was then further refined using homogeneous refinement, global CTF refinement and non-uniform refinement in cryoSPARC. All reported resolutions were determined based on the ‘gold standard’ of 0.143 for the Fourier shell correlation criterion^39^. To model the complexes, our previously published cryo-EM structure of apo DNA-PKcs (PDB, 6ZFP) was used as an initial template for the protein^13^. The template was first rigid-body-fitted into the maps in CHIMERA and CHIMERAX followed by real-space refinement in PHENIX^40–42^. The ligand was then modelled into the corresponding density in an EM map in COOT, followed by further refinement rounds in PHENIX and model building in COOT^42,43^.

### Reporting summary

Further information on research design is available in the Nature Research Reporting Summary linked to this paper.

## Online content

Any methods, additional references, Nature Research reporting summaries, source data, extended data, supplementary information, acknowledgements, peer review information; details of author contributions and competing interests; and statements of data and code availability are available at 10.1038/s41586-021-04274-9.

## Supplementary information

Supplementary Information This file contains Supplementary Tables 1 and 2, Supplementary Fig. 1 and supplementary references.

Reporting Summary

## Data Availability

Cryo-EM maps have been deposited in the Electron Microscopy Data Bank under accession numbers EMDB-13062 (DNA-PKcs in complex with NU7441), EMDB-13064 (DNA-PKcs in complex with ATPγS), EMDB-13067 (DNA-PKcs in complex with wortmannin), EMDB-13068 (DNA-PKcs in complex with AZD7648), EMDB-13069 (DNA-PKcs in complex with M3814) and EMDB-13443 (DNA-PK in complex with ATPγS). Model coordinates have been deposited in the Protein Data Bank under accession numbers 7OTM (DNA-PKcs in complex with NU7441), 7OTP (DNA-PKcs in complex with ATPγS), 7OTV (DNA-PKcs in complex with wortmannin), 7OTW (DNA-PKcs in complex with AZD7648) and 7OTY (DNA-PKcs in complex with M3814). The Hotspots application programming interface (API) is available from https://github.com/prcurran/hotspots under an MIT license, dependent on the commercial CSD Python API.
