# Supplementary material for: Structural insights into inhibitor regulation of the DNA repair protein DNA-PKcs
Source: Nature. 2022 Jan 5;601(7894):643–8. doi: 10.1038/s41586-021-04274-9 (PMC8791830; doi:10.1038/s41586-021-04274-9)
Supplement: Supplementary file 1 — This file contains Supplementary Tables 1 and 2, Supplementary Fig. 1 and supplementary references. [file 41586_2021_4274_MOESM1_ESM.pdf]

nature portfolio

https://doi.org/10.1038/s41586-021-04274-9

Supplementary information

Structural insights into inhibitor regulation of the DNA repair protein DNA-PKcs

In the format provided by the
authors and unedited

Cryo-EM Structural Insights into Inhibitor Regulation of DNA-PKcs

Shikang Liang[1+], Sherine E. Thomas[1], Amanda K. Chaplin[1], Steven W. Hardwick[2], Dimitri Y. Chirgadze [2], Tom L. Blundell[1+]

1. Department of Biochemistry, University of Cambridge, Sanger Building, Tennis Court Road, Cambridge, CB2 1GA, United Kingdom.
2. Cryo-EM Facility, Department of Biochemistry, University of Cambridge, Sanger Building, Tennis Court Road, Cambridge, CB2 1GA, United Kingdom

[+] Joint corresponding authors:

Tom L. Blundell Email: tlb20@cam.ac.uk

Shikang Liang Email: sl744@cam.ac.uk

Table of contents

Supplementary Table 1. Information of the ATP competitive inhibitors.

Supplementary Table 2. Cryo-EM data collection, refinement and validation statistics.

Supplementary Figure 1. Uncropped gel of Extended Data Figure 4d.

Name	Formula	Molecular Weight	2D structure	IC50 (DNA-PK inhibition)
wortmannin	C23H24O8	428.43	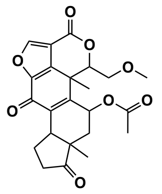	16 nM [1]
NU7441	C25H19NO3S	413.49	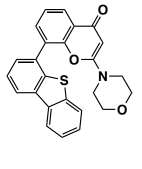	14 nM [2]
AZD7648	C18H20N8O2	380.40	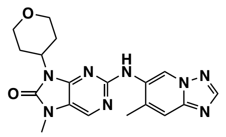	0.6 nM [3]
M3814	C24H21ClFN5O3	481.91	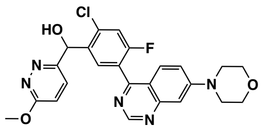	0.6 nM [4]

Supplementary Table 1. Information of the ATP competitive inhibitors.

	DNA-PKcs- NU7441 (EMDB-13062) (PDB 7OTM)	DNA-PKcs-ATPγS (EMDB-13064) (PDB 7OTP)
**Data collection and processing**		
Magnification	130k	130k
Voltage (kV)	300	300
Electron exposure (e–/Å^2)	47.3	46.8
Defocus range (μm)	-0.8 to -2.9	-0.8 to -2.9
Pixel size (Å)	0.652	0.652
Symmetry imposed	C1	C1
Initial particle images (no.)	305666	251481
Final particle images (no.)	115505	63313
Map resolution (Å)	3.33	3.40
FSC threshold	0.143	0.143
Map resolution range (Å)	2.9 to > 10	2.9 to >10
**Refinement**		
Initial model used (PDB code)	6ZFP	6ZFP
Model resolution (Å)	3.33	3.40
FSC threshold	0.143	0.143
Model resolution range (Å)	n/a	n/a
Map sharpening *B* factor (Å^2)	82.7	84.1
Model composition		
Non-hydrogen atoms	29040	29039
Protein residues	3636	3636
Ligands	1	3
B factors (Å^2)		
Protein	68.73	99.18
Ligand	51.36	73.84
R.m.s. deviations		
Bond lengths (Å)	0.009	0.007
Bond angles (°)	0.929	0.851
Validation		
MolProbity score	2.28	2.22
Clashscore	17.66	16.07
Poor rotamers (%)	0.28	0.16
Ramachandran plot		
Favored (%)	90.56	91.17
Allowed (%)	9.22	8.63
Disallowed (%)	0.22	0.19

	DNA-PKcs-wortmannin (EMDB-13067) (PDB 7OTV)	DNA-PKcs-AZD7648 (EMDB-13068) (PDB 7OTW)
**Data collection and processing**		
Magnification	130k	130k
Voltage (kV)	300	300
Electron exposure (e–/Å^2)	47.9	46.8
Defocus range (μm)	-0.8 to -2.9	-0.8 to -2.9
Pixel size (Å)	0.652	0.652
Symmetry imposed	C1	C1
Initial particle images (no.)	340145	539459
Final particle images (no.)	72351	164192
Map resolution (Å)	3.24	2.99
FSC threshold	0.143	0.143
Map resolution range (Å)	2.9 to >10	2.7 to >10
**Refinement**		
Initial model used (PDB code)	6ZFP	6ZFP
Model resolution (Å)	3.24	2.99
FSC threshold	0.143	0.143
Model resolution range (Å)	n/a	n/a
Map sharpening *B* factor (Å^2)	80.7	87.5
Model composition		
Non-hydrogen atoms	29041	29038
Protein residues	3636	3636
Ligands	1	1
B factors (Å^2)		
Protein	114.55	45.13
Ligand	74.85	48.55
R.m.s. deviations		
Bond lengths (Å)	0.008	0.009
Bond angles (°)	1.202	0.935
Validation		
MolProbity score	2.1	2.26
Clashscore	12.03	15.83
Poor rotamers (%)	0.34	0.28
Ramachandran plot		
Favored (%)	91.42	89.87
Allowed (%)	8.47	9.97
Disallowed (%)	0.11	0.17

	DNA-PKcs-M3814<br>(EMDB-13069)<br>(PDB 7OTY)
**Data collection and processing**	
Magnification	130k
Voltage (kV)	300
Electron exposure (e–/Å^2)	47.9
Defocus range (μm)	-0.8 to -2.9
Pixel size (Å)	0.652
Symmetry imposed	C1
Initial particle images (no.)	540811
Final particle images (no.)	209036
Map resolution (Å)	2.96
FSC threshold	0.143
Map resolution range (Å)	2.7 to >10
**Refinement**	
Initial model used (PDB code)	6ZFP
Model resolution (Å)	2.96
FSC threshold	0.143
Model resolution range (Å)	n/a
Map sharpening *B* factor (Å^2)	83.4
Model composition	
Non-hydrogen atoms	29034
Protein residues	3636
Ligands	1
B factors (Å^2)	
Protein	58.32
Ligand	43.67
R.m.s. deviations	
Bond lengths (Å)	0.013
Bond angles (°)	1.070
Validation	
MolProbity score	2.37
Clashscore	20.99
Poor rotamers (%)	0.75
Ramachandran plot	
Favored (%)	89.67
Allowed (%)	10.13
Disallowed (%)	0.19

Supplementary Table 2. Cryo-EM data collection, refinement and validation statistics

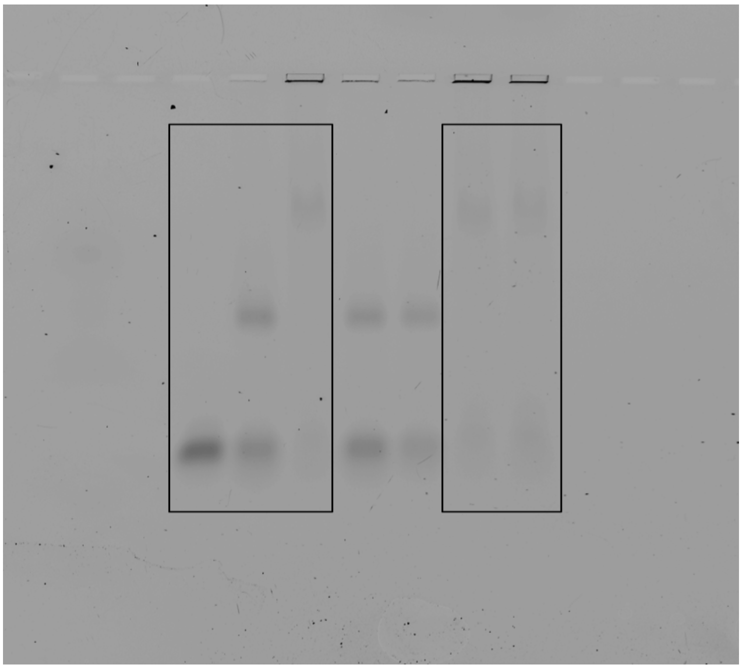

Supplementary Figure 1. Uncropped gel of Extended Data Figure 4d.

References

1. Sarkaria, J. N. *et al.* Inhibition of phosphoinositide 3-kinase related kinases by the radiosensitizing agent wortmannin. *Cancer Res.* **58**, 4375–4382 (1998).
2. Leahy, J. J. J. *et al.* Identification of a highly potent and selective DNA-dependent protein kinase (DNA-PK) inhibitor (NU7441) by screening of chromenone libraries. *Bioorganic Med. Chem. Lett.* **14**, 6083–6087 (2004).
3. Goldberg, F. W. *et al.* Abstract DDT01-02: Discovery and first structural disclosure of AZD7648: A potent and selective DNA-PK inhibitor. in *Cancer Research* **79**, DDT01-02-DDT01-02 (American Association for Cancer Research (AACR), 2019).
4. Zenke, F. T. *et al.* Pharmacologic inhibitor of DNA-PK, M3814, potentiates radiotherapy and regresses human tumors in mouse models. *Mol. Cancer Ther.* **19**, 1091–1101 (2020).
