# Supplementary material for: Structural insights into inhibitor regulation of the DNA repair protein DNA-PKcs
Source: Nature. 2022 Jan 5;601(7894):643–8. doi: 10.1038/s41586-021-04274-9 (PMC8791830; doi:10.1038/s41586-021-04274-9)
Supplement: Supplementary file 2 — Reporting Summary [file 41586_2021_4274_MOESM2_ESM.pdf]

nature portfolio

Corresponding author(s): Tom Blundell, Shikang Liang

Last updated by author(s): Nov 8, 2021

Reporting Summary

Nature Portfolio wishes to improve the reproducibility of the work that we publish. This form provides structure for consistency and transparency in reporting. For further information on Nature Portfolio policies, see our Editorial Policies and the Editorial Policy Checklist.

Please do not complete any field with "not applicable" or n/a. Refer to the help text for what text to use if an item is not relevant to your study.
For final submission: please carefully check your responses for accuracy; you will not be able to make changes later.

Statistics

For all statistical analyses, confirm that the following items are present in the figure legend, table legend, main text, or Methods section.

n/a	Confirmed	
☐	☒	The exact sample size (*n*) for each experimental group/condition, given as a discrete number and unit of measurement
☐	☒	A statement on whether measurements were taken from distinct samples or whether the same sample was measured repeatedly
☒	☐	The statistical test(s) used AND whether they are one- or two-sided<br>*Only common tests should be described solely by name; describe more complex techniques in the Methods section.*
☒	☐	A description of all covariates tested
☒	☐	A description of any assumptions or corrections, such as tests of normality and adjustment for multiple comparisons
☒	☐	A full description of the statistical parameters including central tendency (e.g. means) or other basic estimates (e.g. regression coefficient) AND variation (e.g. standard deviation) or associated estimates of uncertainty (e.g. confidence intervals)
☒	☐	For null hypothesis testing, the test statistic (e.g. *F*, *t*, *r*) with confidence intervals, effect sizes, degrees of freedom and *P* value noted<br>*Give P values as exact values whenever suitable.*
☒	☐	For Bayesian analysis, information on the choice of priors and Markov chain Monte Carlo settings
☒	☐	For hierarchical and complex designs, identification of the appropriate level for tests and full reporting of outcomes
☒	☐	Estimates of effect sizes (e.g. Cohen's *d*, Pearson's *r*), indicating how they were calculated

Our web collection on statistics for biologists contains articles on many of the points above.

Software and code

Policy information about availability of computer code

Data collection	Thermo-Scientific EPU 2.10.0
Data analysis	WARP v1.0.8, cryoSPARC v2.5.0, Phenix v1.19, COOT 0.8.9.2, Chimera 1.15.0, ChimeraX-1.1.1

For manuscripts utilizing custom algorithms or software that are central to the research but not yet described in published literature, software must be made available to editors and reviewers. We strongly encourage code deposition in a community repository (e.g. GitHub). See the Nature Portfolio guidelines for submitting code & software for further information.

Data

Policy information about availability of data

All manuscripts must include a data availability statement. This statement should provide the following information, where applicable:
- Accession codes, unique identifiers, or web links for publicly available datasets
- A description of any restrictions on data availability
- For clinical datasets or third party data, please ensure that the statement adheres to our policy

Cryo-EM maps have been deposited in the Electron Microscopy Data Bank under accession numbers EMD-13062 (DNA-PKcs in complex with NU7441), EMD-13064 (DNA-PKcs in complex with ATPγS), EMD-13067 (DNA-PKcs in complex with wortmannin), EMD-13068 (DNA-PKcs in complex with AZD7648), EMD-13069 (DNA-PKcs in complex with M3814) and EMD-13443 (DNA-PK in complex with ATPγS). Model coordinates have been deposited in the Protein Data Bank under accession numbers 7OTM (DNA-PKcs in complex with NU7441), 7OTP (DNA-PKcs in complex with ATPγS), 7OTV (DNA-PKcs in complex with wortmannin), 7OTW (DNA-PKcs in complex with AZD7648) and 7OTY(DNA-PKcs in complex with M3814). The model referenced for model building (6ZFP) is available in the Protein Data Bank. The Hotspots API is available from https://github.com/prcurran/hotspots under the MIT license, dependent upon the commercial CSD Python API.

Field-specific reporting

Please select the one below that is the best fit for your research. If you are not sure, read the appropriate sections before making your selection.

☒ Life sciences ☐ Behavioural & social sciences ☐ Ecological, evolutionary & environmental sciences

Life sciences study design

All studies must disclose on these points even when the disclosure is negative.

Sample size	There were no statistical methods used to predetermine sample size. Each cryo-EM dataset was collected independently. The final micrograph and particle numbers are comparable to recent cryo-EM studies in the field for the final reconstruction.
Data exclusions	During the cryo-EM data analysis, bad particles and contamination were discarded through iterative 3D classifications. This procedure is standard in the single particle analysis cryo-EM field.
Replication	The attempts at replication of sample preparation and DNA-PKcs/ligand complex reconstruction for cryo-EM investigation and EMSA were successful.
Randomization	Randomization was not relevant to our study since no clinical trials or drug treatment assays were performed.
Blinding	Blinding was not relevant to our study since no clinical trials or drug treatment assays were performed.

Reporting for specific materials, systems and methods

We require information from authors about some types of materials, experimental systems and methods used in many studies. Here, indicate whether each material, system or method listed is relevant to your study. If you are not sure if a list item applies to your research, read the appropriate section before selecting a response.

Materials & experimental systems

n/a	Involved in the study	
☒	☐	Antibodies
☒	☐	Eukaryotic cell lines
☒	☐	Palaeontology and archaeology
☒	☐	Animals and other organisms
☒	☐	Human research participants
☒	☐	Clinical data
☒	☐	Dual use research of concern

Methods

n/a	Involved in the study	
☒	☐	ChIP-seq
☒	☐	Flow cytometry
☒	☐	MRI-based neuroimaging

This checklist template is licensed under a Creative Commons Attribution 4.0 International License, which permits use, sharing, adaptation, distribution and reproduction in any medium or format, as long as you give appropriate credit to the original author(s) and the source, provide a link to the Creative Commons license, and indicate if changes were made. The images or other third party material in this article are included in the article's Creative Commons license, unless indicated otherwise in a credit line to the material. If material is not included in the article's Creative Commons license and your intended use is not permitted by statutory regulation or exceeds the permitted use, you will need to obtain permission directly from the copyright holder. To view a copy of this license, visit http://creativecommons.org/licenses/by/4.0/
